# Current and future perspectives on lumbar degenerative disc disease: a UK survey exploring specialist multidisciplinary clinical opinion

**DOI:** 10.1136/bmjopen-2016-011075

**Published:** 2016-09-15

**Authors:** Janet A Deane, Alison H McGregor

**Affiliations:** MSK Lab, Imperial College London, London, UK

**Keywords:** Low Back Pain, Clinical Interpretation, Lumbar Degenerative Disc Disease, Lumbar Disc Degeneration, Recurrent Pain, Degenerative Disc Disease

## Abstract

**Objectives:**

Despite lumbar degenerative disc disease (LDDD) being significantly associated with non-specific low back pain and effective treatment remaining elusive, specialist multidisciplinary clinical stakeholder opinion remains unexplored. The present study examines the views of such experts.

**Design:**

A reliable and valid electronic survey was designed to establish trends using theoretical constructs relating to current assessment and management practices. Clinicians from the Society of Back Pain Research (SBPR) UK were invited to take part. Quantitative data were collated and coded using Bristol Online Surveys (BOS) software, and content analysis was used to systematically code and categorise qualitative data.

**Setting:**

Specialist multidisciplinary spinal interest group in the UK.

**Participants:**

38/141 clinically active, multidisciplinary SBPR members with specialist spinal interest participated. Among them, 84% had >9 years postgraduate clinical experience.

**Interventions:**

None.

**Outcome measures:**

Frequency distributions were used to establish general trends in quantitative data. Qualitative responses were coded and categorised in relation to each theme and percentage responses were calculated.

**Results:**

LDDD symptom recurrence, in the absence of psychosocial influence, was associated with physical signs of joint stiffness (26%), weakness (17%) and joint hypermobility (6%), while physical factors (21%) and the ability to adapt (11%) were postulated as reasons why some experience pain and others do not. No one management strategy was supported exclusively or with consensus. Regarding effective modalities, there was no significant difference between allied health professional and medic responses (p=0.1–0.8). The future of LDDD care was expressed in terms of improvements in patient communication (35%), patient education (38%) and treatment stratification (24%).

**Conclusions:**

Results suggest that multidisciplinary expert spinal clinicians appear to follow UK-based assessment guidelines with regard to recurrent LDDD; there are, however, inconsistencies in the management approaches supported. This reflects the current literature and the lack of specific, formalised guidance. LDDD treatment stratification and further research are explicitly supported.

**Strengths and limitations of this study**

- This study is an online questionnaire survey designed to explore current perspectives of degenerative lumbar disc disease from a clinical spinal specialist interest group (Society of Back Pain Research, UK).
- The survey was designed specifically to explore current trends with regard to the following theoretical constructs: training and education, general knowledge, assessment and management practices and future directions.
- This study suggests that experienced clinicians follow an evidence-based approach with regard to assessment; however, no one management strategy is supported with consensus, which reflects the current literature.
- Treatment stratification and exploration of the biological markers are explicitly supported.
- Data are from a selected sample of clinically active, experienced health professionals with a specialist interest in the spine within the UK, which limits the generalisability of results.

## Introduction

Low back pain (LBP) is the top global cause of years lived with disability.^1^ The majority of LBP is classified as non-specific LBP (NSLBP), affecting 30% of the UK population annually.^2^ Over the past 25 years, research has improved the treatment of NSLBP through activity promotion, increasing numbers of treatment-focused randomised controlled trials (RCTs) and the inclusion of a biopsychosocial approach to therapy.^3–5^

However, the results of RCTs focusing on varied treatment approaches for NSLBP are limited by factors such as sample size, the lack of a control group, the heterogeneity of the population under investigation, treatment fidelity and non-specific treatment effects, such as support or empathy of the treatment provider.^6^ In fact, to date there seems to be little appreciable benefit, with current treatments offering small to moderate effects in terms of a sustained improvement in the quality of life and disability.^7–9^

Biopsychosocial approaches have shown a similar trend. Focus on the psychosocial component through cognitive–behavioural approaches and functional restoration programmes, while seemingly demonstrating the potential to reduce National Health Service (NHS) treatment costs,^10^ have resulted in, at best, moderate treatment effects for patients with NSLBP.^11–15^ Therefore, perhaps it is time to recognise the biological component of the biopsychosocial model in patients with NSLBP, which may permit effective phenotyping of such individuals so that specific stratified treatment approaches may be employed to better effect.

Patients with recurrent NSLBP commonly seek care from multidisciplinary primary and secondary care settings. In order to reduce the ‘fragmentation’ of care, clinical guidelines are used to standardise management based on the best available evidence,^16^ to narrow the gap between ‘best’ and ‘usual’ practice^17^ and control for differences in training, knowledge and scope of the team of disciplines involved.^18^ National and international guidelines have been established to standardise recurrent NSLBP assessment and management and generally concur in terms of recommendations relating to diagnostic triage, assessment, activity promotion and recognition of psychosocial factors; however, management discrepancies exist, particularly in terms of exercise prescription, spinal manipulation and patient information.^4^ These discrepancies may be due to a lack of strong evidence and recommendations based on consensus and discussion where evidence is lacking.

The recent publication of multidisciplinary guidelines from the National Institute for Health and Care Excellence (NICE, UK) reflects this.^19^ The NSLBP management recommendations within these guidelines are not explicit and assume unlimited NHS resource: promoting self-management, staying active, education, structured exercise (up to 8 sessions over up to 12 weeks), manual therapy including spinal manipulation (9 sessions for up to 12 weeks), acupuncture (for 10 sessions for up to 12 weeks) and, if improvements are unsatisfactory, referral for combined physical and psychological treatment (100 hours over 8 weeks).^2^ ^19^ Although such multidisciplinary guidelines are justifiably limited, given the available evidence and the current climate of austerity (where, in the UK, the number of treatment sessions may be restricted to three or four in private or public healthcare^20^), perhaps it is time to consider a more specific, realistic and practical way forward so that implementation is possible. Current clinical and patient opinion reflects this need.^20^ ^21^

In the absence of psychosocial influence, it remains challenging to manage recurrent NSLBP symptoms and to guide effective healthcare provision in this area, with most clinicians justifiably favouring a suboptimal ‘one size fits all approach’^22^ in the absence of a suitable alternative. However, recent NSLBP research supports a system of subclassification through which targeted treatments have been successfully employed.^23^ This would seem to indicate that perhaps a more stratified and specific approach is sensible.

Lumbar degenerative disc disease (LDDD) is a condition which has been found to be significantly associated (p<0.001) with NSLBP, the lifetime prevalence of which may be as much as 80% with an annual prevalence rate of 25–60%.^24^ LDDD describes a set of signs of disc degeneration and associated symptoms, namely dis-ease or pain. Although disc degeneration is often associated with LBP,^25–27^ it is not always synonymous with LBP, occurring in symptomatic and asymptomatic populations.^28^

The presence or absence of pain with LDDD provides a unique opportunity to examine the differences between those with LDDD and pain and those without in order to understand and potentially subclassify this group according to biological and psychosocial markers.

Although clinicians are one of the primary stakeholders in patient care, until now, their views have not been considered with regard to effective LDDD assessment, management and future directions. In order to advance subclassification and treatment stratification of conditions significantly associated with NSLBP, it seems appropriate and timely to gain an appreciation of current knowledge and practice.

This preliminary exploratory work aims to establish current trends in opinion from a multidisciplinary spinal interest group, the Society of Back Pain Research (SBPR) UK. It is hypothesised that clinicians with such specialist interest and expertise in the spine will offer a unique and honest insight into current clinical assessment and management practices, serving to inspire and inform future work.

## Method

### Participants

The study population was defined as clinically active members of the SBPR UK from diverse healthcare settings and health professions.

### Web survey development and delivery

An electronic survey was designed to establish current trends with regard to the following theoretical constructs: training and education, general knowledge relating to LDDD and assessment and management practices. LDDD was defined as lumbar disc degeneration with recurrent pain (>3 months duration).

To ensure that the validity, reliability and respondent satisfaction were maximised, the questionnaire was designed to include simple, objective questions with logical section headers.^29–32^ To reduce bias secondary to the error of omission, the response ‘other’ was included as an option and qualitative comment was invited in relation to specific questions to enhance interpretation.

The electronic survey was conducted between 6 February 2014 and 30 May 2014. The survey was distributed by the SBPR to their specialist interest group members who were invited to take part (Ethics REC reference number: 13/LO/0793). Reminders were sent to non-respondents at 1 and 2 months. A due date was specified on invitation. Informed consent was sought from each participant.

### Face and content validity

To ensure that the content of the questionnaire was meaningful and representative, the survey was developed following a review of the literature. A pilot study involving five independent experts in the spinal field (one professor, one consultant musculoskeletal radiologist, two advanced physiotherapy spinal practitioners and one consultant spinal surgeon) was then used to establish face validity. Following the receipt of feedback, questions were further modified to avoid leading questions and to enhance clarity.

### Intrarater reliability

In order to establish intrarater reliability, 10 chartered physiotherapists with advanced musculoskeletal knowledge were invited to complete the survey at weeks 1 and 3.

To ensure that the results were consistent, quantitative responses for weeks 1 and 3 were coded using the Bristol online survey tool and analysed to determine intrarater reliability using IBM SPSS Statistics Software V.22 (IBM, Armonk, New York, USA). Intrarater reliability in this instance was deemed to be substantial (using Landis and Koch definition,^33^ κ=0.6).

### Data analysis

Descriptive statistics involving frequency distributions were used to establish general trends in opinion. Quantitative data were coded as described above. Mann Whitney U tests for non parametric data (two tailed) were used to establish significant differences between medical professions with regard to LDDD assessment and management confidence and LDDD treatment scores. Normality was evaluated using histograms and the Shapiro Wilks test. A result was considered statistically significant at the 5% level (p≤0.05).

Content analysis was used to objectively and systematically categorise and quantify qualitative data in order to permit successful analysis.^34^ Using an inductive approach, all qualitative responses were read to gain an appreciation of content and context. The recommended organisational phases of coding, grouping, categorisation and abstraction were then employed^35^ to condense qualitative information. To control for the subjective and interpretative process, three health professionals with expertise in the area were invited to review all meaning units, codes, subthemes and themes in conjunction with qualitative responses. Following discussion and reflection, minor amendments were necessary to exclude responses that were deemed ‘too general to code’. Decisions were made by consensus.

## Results

### Response rate and demographics

All quantitative and qualitative responses (response rate 38/141, 27%) were collated and analysed using Bristol Online Surveys (BOS) software (University of Bristol). Respondents were SBPR members (87% of respondents graduated within the UK), clinically active and represented a variety of clinical backgrounds (nursing (n=1), rheumatology (n=1), physiotherapy (n=22), surgery (n=10) and general practice (n=4)). The majority had >9 years of postgraduate clinical experience (84%) (table 1).

**Table 1:** Respondent interpretations of themes relating to training and education, LDDD definition, impact and future management

Themes	Items	Descriptors	Responses (n)	Response rate (%)
Training and education	Years of postgraduate experience	0–2 years	1	2.6
		3–5 years	0	0
		6–8 years	5	13.2
		9+ years	32	84.2
	Country of graduation	UK	33	86.8
		Other	5	13.2
Definition of LDDD	Definition of LDDD	Dehydrated disc	17	44.7
		Change in disc integrity	26	68.4
		Intervertebral changes	7	18.4
		Disc height reduction	7	18.4
		Symptomatic	13	34.2
		Asymptomatic	4	28.9
		Multifactorial causes	19	50.0
		Do not use this term	2	5.3
		Not a disease	7	18.4
	LDDD prevalence in clinic	0–10%	5	13.2
		10–30%	9	23.7
		30–50%	5	13.2
		50% +	14	36.8
		Unsure	1	2.6
		Not applicable	4	10.5
	LDDD cause	Genetics	13	33
		Posture	6	15
		Movement patterns	7	18
		Smoking	9	23
		Unsure	2	4
		Other (comorbidities)	8	21
	Signs associated with LDDD	Weakness	6	17
		Joint hypermobility	2	6
		Joint stiffness	10	26
		Pain	11	30
		Paraesthesia	5	14
		Unsure	2	4
		Other (stenosis, spondylolisthesis, reduced lordosis)	4	10
LDDD assessment	Confirmation of diagnosis	MRI	36	94.7
		Physical assessment	21	55.3
		None of the above	0	0
		Other (medical history)	17	44.7
	MRI findings are associated with LDDD	Osteophytes	25	65.8
		Annular tear	28	73.7
		Disc bulges	32	84.2
		Disc herniations	28	73.7
		Reduction in disc height	35	92.1
		Evidence of disc dehydration	34	90
		Unsure	1	2.6
		Other (Modic or end plate changes)	12	31.6
	Classification system used for grading LDDD	Modic grading system	18	47.4
		Pfirrmann grading system	9	23
		Modified Pfirrmann grading	2	5.3
		None of the above	10	26.3
		Other (do not use classification systems)	7	18.4
	Why degenerative change is often not proportional to presenting symptoms	Pyschosocial factors	9	23
		Pain perception/interpretation	5	13
		Pain mechanisms	6	16
		Physical factors	8	21
		Ability to adapt	4	11
		Infection	1	3
		Genetics	1	3
		Don't know	6	16
		DLDD is not the cause	3	8
	Use of functional tests as part of assessment	Yes	34	89.5
		No	4	10.5
	Most effective functional tests used in LDDD assessment	Gait	19	50
		Double leg stand (eyes open and eyes shut)	6	15.8
		Single leg stand (eyes open and eyes shut)	8	21.1
		Double leg squat	5	13.2
		Single leg squat	6	15.8
		Lunge	3	7.9
		Sit to stand	14	36.8
		All of the above	4	10.5
		None of the above	5	13.2
		Other (repeated movement and range of movement)	13	34.2
	Muscles most commonly affected	Calf	1	2.6
		Ankle dorsiflexors	1	2.6
		Quads	1	2.6
		Hamstrings	1	2.6
		Gluteus medius	4	10.5
		Gluteus maximus	4	10.5
		Extensors	10	26.3
		Erector spinae	10	26.3
		Multifidus	15	39.5
		Abdominals	4	10.5
		TVA	7	18.4
		Psoas	1	2.6
		Quadratus lumborum	2	5.3
		Lat dorsi	1	2.6
		Unsure	5	13.2
LDDD impact and future	Impact of LDDD with recurrent pain on quality of life	Serious	6	15.8
		Significant	22	57.9
		Minimal	2	5.3
		None	0	0
		Unsure	8	21.1
	Impact of psychosocial factors on LDDD and recurrent pain	Serious	8	21.1
		Significant	25	65.8
		Minimal	1	2.6
		None	0	0
		Unsure	4	10.5
	Future improvements in care	Inclusive communication	13	35.1
		Stratified treatment	9	24.3
		Effective patient education	14	37.8
		Encourage self-management	4	10.8
		Realistic goals and expectations	3	8.1
		Evidence-based management	9	24.3
		Consideration of long term	1	2.7
		Holistic approach	3	8.1
		Early intervention and service access	5	13.5
		Specific diagnosis	1	2.7
		MDT approach	2	5.4

LDDD, lumbar degenerative disc disease; MDT, multidisciplinary team; TVA, transversus abdominus.

### LDDD: definition, prevalence, signs and causes

Most clinicians (37%) reported a high LDDD prevalence (50% or more) from their clinical experience of working in primary and secondary care settings. The primary causal factors for LDDD were cited as genetics (33%), posture (15%) and movement patterns (18%) (smoking, age, obesity, occupation and previous trauma were also referenced).

Clinicians defined the signs and symptoms associated with LDDD as being dependent on whether the condition was acute (<3 months duration) or a long-standing, recurrent presentation (>3 months duration); reporting pain as the dominant symptom in long-standing degenerative (30%) scenarios. The clinical signs reported to most likely result in recurrence included joint stiffness (26%), weakness (17%) and joint hypermobility (6%) (table 1).

### LDDD assessment and management

Of the total respondents, 47–50% reported confidence levels of eight or over with regard to LDDD assessment and management (where 0/10 represented no confidence and 10/10 representing extreme confidence). No significant difference in clinical confidence rating was found between allied health professionals (AHPs) and medics (p=0.8–0.9; table 2).

**Table 2:** AHP and medic mean clinical confidence and LDDD treatment efficacy scores

Themes	AHP mean scores (SD)	Medic mean scores (SD)	p Value
Confidence (where 0 is not confident and 10 is confident)			
Assessment confidence	7.2 (2.2)	5.5 (1.5)	0.8
Management confidence	7.2 (2.4)	7 (1.5)	0.9
Treatment efficacy (where 3 is effective and 1 is ineffective)			
Education and reassurance	2.9 (0.6)	2.8 (0.4)	0.2
Acupuncture	1.3 (0.9)	1.5 (0.7)	0.8
Core stability training	2.7 (0.5)	2.2 (0.5)	0.1
Manual therapy	2.3 (0.5)	1.6 (0.8)	0.02*
Cognitive–behavioural approach	2.6 (0.4)	2.4 (0.4)	0.4
Pain management	2.5 (0.5)	2.3 (0.6)	0.2
Electrotherapy	1.5 (0.6)	0.7 (0.3)	0.03
Surgery	1.5 (0.7)	2.1 (0.7)	0.2
Classes/groups	2.3 (0.5)	2.4 (0.6)	0.8

*Statistical significance at the 5% level (p≤0.05).

AHP, allied health professionals; LDDD, lumbar degenerative disc disease.

The majority of respondents considered MRI (95%) and physical assessment (55%) to be the most important clinical tools when confirming a diagnosis of LDDD. Respondents considered a reduction of disc height (92%) and disc dehydration (90%) as the most important variables when diagnosing LDDD (table 1).

In terms of grading LDDD on MRI, 47% of respondents use the Modic classification system clinically to grade degenerative end plate or Modic changes, which are significantly associated with LDDD, while 23% and 5% used disc-specific grading tools such as the Pfirrmann and the modified Pfirrmann grading tools, respectively.

Ninety per cent of respondents use functional movements as part of routine assessment, of which the most commonly assessed movements include gait (50%), sit to stand (37%), double leg standing (eyes open and shut) (16%) and single leg standing (eyes open and shut) (21%) with muscles most commonly affected by the condition reported to include the erector spinae (26%) and multifidus (40%) and to a lesser degree the abdominals and gluteals. Only one respondent cited thigh or shank muscle involvement.

**Figure 1:**
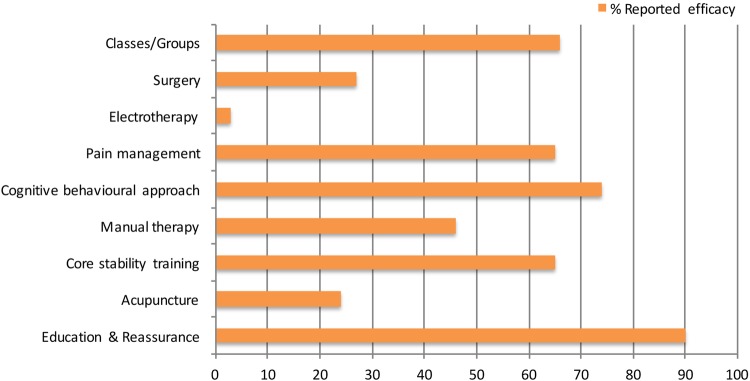
The modalities which respondents cite as the most effective for managing LDDD. LDDD, lumbar degenerative disc disease.

In terms of recurrent, long-standing effective LDDD management, there was no significant difference between AHP and medic responses (p=0.1–0.8; table 2). Education and reassurance, pain management, cognitive–behavioural therapy, core stability training and group approaches to treatment were reported to be the most effective (figure 1) with Pilates, yoga, pain management and back to fitness programmes being favoured over a hands-on approach. Although significant differences in mean scores were noted between AHP and medics in relation to manual therapy (p=0.02) and electrotherapy (p=0.03), the mean scores reflect AHP and medic interpretations that electrotherapy and manual therapy are ‘not very effective’ or ‘ineffective’, respectively (table 2). This difference may represent a professional bias in terms of the profession-specific treatments each employ; however, scores indicate that both groups interpret such modalities to be suboptimal.

Recurrence was also reported to significantly affect quality of life (58%), with psychosocial factors (66%) significantly influencing LDDD symptom recurrence.

### Qualitative data

Respondents were asked to define LDDD to establish the meaning they attributed to this term. The majority defined this condition in terms of disc dehydration (45%) with concomitant changes in disc height (19%), disc integrity (68%) and the vertebrae (18%). Forty-five per cent of respondents also referred to an associated presence (34%) and/or absence (11%) of symptoms. The multifactorial nature of the condition (with reference to ageing, environmental and genetic influences) was reported by 50% of respondents. Interestingly, 18% of respondents did not feel that the diagnostic term LDDD is useful as it is ‘not a disease’, with 5% reporting that they therefore do not use the term.

**Figure 2:**
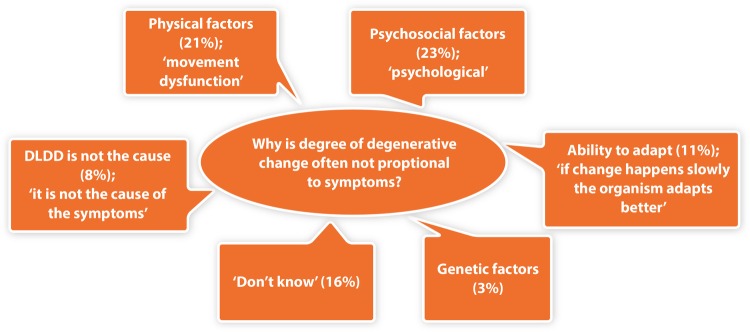
Respondent interpretations of why the degree of degenerative change associated with LDDD is often not proportional to the presenting symptoms. LDDD, lumbar degenerative disc disease.

The current understanding relating to the apparent mismatch between the degree of pain patients may or may not experience and MRI indicators of lumbar disc degeneration was also explored. In the absence of psychosocial influence, this phenomenon was explained by physical factors (21%, including movement dysfunction, muscle activation and strength) and the ability to physically adapt (11%; figure 2). Psychosocial factors (23%), pain perceptions and interpretations (13%) and the complexity of pain mechanisms (16%) were also reported. Sixteen per cent did not understand why this phenomenon occurs, with 8% of respondents believing LDDD is not causal, implying that it may result from pain.

**Figure 3:**
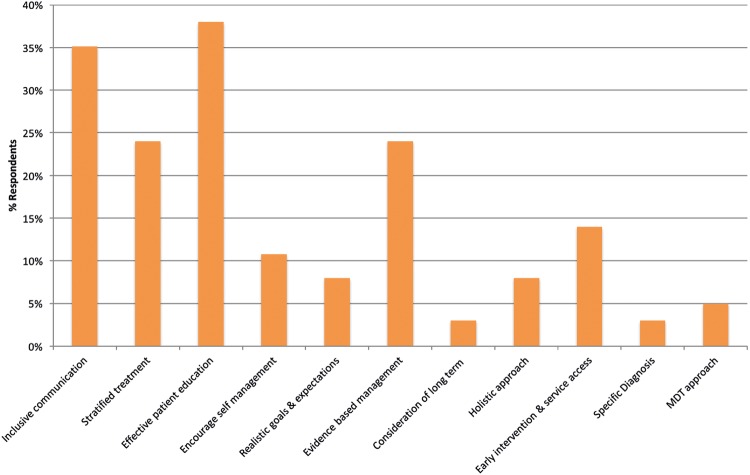
The variety of ways in which respondents believe LDDD management can be improved in the future. LDDD, lumbar degenerative disc disease.

The future management of recurrent and long-standing LDDD was seen by the majority of respondents in terms of inclusive communication (35%) and patient education (38%; figure 3). The concept of treatment stratification (24%) and the emphasis placed upon advancing evidence-based management practices (24%) through research were recommended. This question was unanswered by one respondent and the missing response was not replaced by imputed values.

## Discussion

This exploratory study presents novel data; for the first time, the frank opinions of multidisciplinary clinicians have been explored, with responses that are based on clinical experience, thereby representing clinical practice. In the absence of definitive answers with regard to LDDD assessment and management of recurrent symptoms, clinical decisions have to be made. The results from our study represent an evidence-informed approach, where a wealth of clinical experience and insight is presented in conjunction with the current evidence and practice.

### LDDD assessment

As a result of our exploratory work, it is clear that the results obtained reflect the views of experienced and knowledgeable clinicians, in that the majority of respondents reported a high level of LDDD assessment and management confidence and attributed the same meaning to the condition, in terms of definition, as the authors did. This was further enhanced through reliability and validity testing of the survey prior to distribution.

It is of interest that when clinicians were asked how they define LDDD, 18% cited that they do not make use of the term LDDD: ‘I do not consider it to be a disease’, with 5% responding that they simply did not use the term. There were also repeated themes referenced in relation to diagnosis; on the one hand, respondents proposed avoiding labelling; ‘stop interfering and making life worse…avoid labeling’, while, on the other, a more specific diagnosis than NSLBP was requested: ‘I think we need to give patients a diagnosis that is more specific than NSLBP’. While as a set of signs and symptoms, the term LDDD seems representative and encompassing, for the clinician, it seems a limited term; having potential to engender fear, while alternatives such as NSLBP fail to offer reassurance to patients who fear judgement and stigmatisation from this generic, ‘non’-label.^21^ ^36^ Clearly further clarification and guidance is required.

SBPR clinicians agreed that LDDD is highly prevalent (50% or more), which is reflective of the environments in which clinicians with specialist spinal interest work. However, reported prevalent rates vary within the literature as assessment gold standards are lacking and population definitions remain inconsistent. Estimates therefore range between 40% and 90% in symptomatic^26^ and 10–80% in asymptomatic populations.^37^

The gold standard for assessment of structural disc degeneration is MRI.^38^ In order to classify disc degeneration, several reliable and discriminatory methods are reported, including the Pfirrmann and the modified Pfirrmann grading systems. Modic or vertebral end plate changes^39^ are also commonly used by radiologists due to their significant association with NSLBP^40^ and significant correlation with degenerative disc grading (Pfirrmann and modified Pfirrmann grades (p<0.01)).^41^ In the current study, clinicians recognise MRI as the assessment gold standard, with the majority favouring Modic classification and to a lesser degree Pfirrmann classification systems. This is not surprising as the Modic system is easy to apply, score and shows a high degree of association with degenerative change unlike the Pfirrmann system, which relies on multiple gradings, descriptors and images.

The reported causal factors cited by clinicians concur with the literature and it is evident that, from a clinical viewpoint, the effect of posture and movement patterns should not be underestimated and that weakness, joint stiffness and hypermobility may all play a part in the recurrence and longevity of LDDD symptoms. Indeed, in the section of the questionnaire that referred to physical assessment, clinicians regarded functional physical assessment as key; the majority tending to assess movements typically associated with the exacerbation of symptoms and which are, therefore, more meaningful to patients (eg, gait or sit to stand). However, it is interesting that clinicians do not standardly consider the influence of the whole kinematic chain (ie, spine, pelvis and lower limb) in terms of its contribution towards recurrence, focusing on specific muscles such as the erector spinae and transversus abdominus. If clinical emphasis is to successfully change from psychosocial to biopsychosocial, in order to effectively stratify treatment, we need to avoid narrowing our hypothesis too early and consider the global biological chain of contributing factors.

In the assessment section, clinicians were asked why they felt LDDD symptoms were often not found to be proportional to the degree of degeneration evident on MRI. While responses emphasised psychosocial factors and the complexity of pain mechanisms, biological factors such as movement dysfunction, movement strategy and the ability to physically adapt were also supported. However, interestingly 16% of clinicians did not feel that they were equipped with the answers to this question, implying that there is a lot we do not know; ‘the reason is not completely clear’.

### LDDD management

Our results suggest that current specialist practice supports a multimodal, generic approach, which falls in line with current NICE guidelines: promoting self-management, staying active, education, structured exercise, manual therapy including spinal manipulation or referral for combined physical and psychological treatment.^2^ From our research, it would seem that no one strategy is supported exclusively or with consensus. Indeed, from the qualitative responses received, there seems to be confusion regarding the specifics of best practice especially in terms of exercise. Although the authors do not advocate completely defining practice, as clinicians require autonomy in order to be able to make the best clinical judgments, management guidelines which recommend ‘manual therapy’ for nine sessions for up to 12 weeks or ‘structured exercise’ for eight sessions for up to 12 weeks become meaningless to the NHS clinician who is guided to implement a therapy that has not been defined and is unable to fulfil the brief due the cost incurred. Therefore, redefining guidance in order to reflect current practice will be necessary moving forward in order to ensure successful implementation and improve patient outcomes.

Over the years RCTs have focused on an infinite number of intervention combinations, the way treatment is delivered and the cost implications.^10^ ^42–50^ The NICE guidelines^2^ ^19^ outline the best evidence resulting from such trials and the current practice of SBPR clinicians reflects this approach. However, the effect sizes in these trials are at best small to moderate,^9^ implying that we have not found an acceptable solution.

There is no doubt that over the past 25 years, recognition of the psychosocial has improved practice.^5^ Indeed, respondents in this study continue to view the contribution of psychosocial factors as having a significant impact on LDDD symptom recurrence (66%). However, in spite of this, treatments that focus on the psychosocial have been found to have moderate effects.^11^ ^13–15^ In fact, patients with NSLBP have not been found to have significantly higher psychosocial comorbidity than the average patient consulting primary care.^15^

Therefore, it would seem that the ‘back pain revolution’,^51^ which has seemingly absolved clinicians of responsibility (patients with yellow flags or psychosocial factors being referred for group intervention)^52^ and has given the NHS an opportunity to deal with NSLBP in a more ‘cost-effective’ manner, cannot deliver effectively if the clinical over-reliance on the psychosocial continues.

Although classes and group treatments may have their place, the future management of recurrent and long-standing LDDD was seen by the majority of SBPR respondents not only in terms of inclusive communication and patient education but also in terms of treatment stratification and advancing evidence-based management practices.

In relation to communication and patient education, the support of clear, open, honest, collaborative, demedicalised communication in our study is nothing new. However, it is of interest that clinicians feel that this area remains a cause for concern. Given current evidence and guidance, it is possible that being honest is difficult; if one is not guided as to how to best treat, perhaps one is forced to opt for treatment modalities which are, at best, mildly to moderately effective.

The continuation of realistic goal and expectation setting, multidisciplinary support and self-management promotion is supported. However, there is also support for early and prophylactic treatment: ‘Press for greater allocation of resources to treat patients earlier in the process’, and consideration of the long term: ‘advise and support patients in the long term and review them regularly as opposed to discharging them if immediate results are not obtained’. Advancing the clinical evidence base in the area of management and stratification of treatment were also cited as ways of improving the future of care: ‘is there a modality that is best for the patients and can we sub-classify these patients to ensure they are getting the best care?’, ‘we need to offer specific treatment’, ‘set realistic not blinkered system approach to exercise’. This area clearly requires investigation.

The requirement for specialist spinal knowledge and experience from clinically active health professionals, is a strength in this study. The response rate of 27% for this exploratory work is over double the average cited for web surveys (11%);^53^ however, due to the specific inclusion criteria employed and limited accessibility to groups of such specific clinical interest in the UK, it is recognised that the sample size limits the generalisability of the results. Assumptions made regarding honesty and accuracy of responses, potential non-response bias and convenience sampling, which affect most studies of this nature, may also be regarded as limitations.

## Conclusions

This survey provides novel information relating to LDDD and the perspectives of multidisciplinary clinicians in the UK with a specialist spinal interest. Although the aim of this study is not to make definitive recommendations, it seems reasonable to acknowledge the experienced multidisciplinary voice, whose daily work in environments requires decisions to be made in the absence of certainty or guidelines that provide the definitive answer. Regarding how we as health professionals may seek to improve the future of management of this condition, there is a clear message; transparent patient communication is required as there is a lot we have yet to understand. Expert clinicians are also keen to allocate resources to treat patients earlier in the process and to review patients regularly without defined discharge so that patients are empowered to self-manage without fear of being abandoned by the system. Finally, there is a commitment to tailored, evidence-based management, to suit the individual on a holistic level. Moving forward, the challenge will be to develop defined clinical subgroups for which effective intervention is possible.
